# Differential Expression of Inflammation-Related Genes in Down Syndrome Patients with or without Periodontal Disease

**DOI:** 10.1155/2019/4567106

**Published:** 2019-10-21

**Authors:** M. Baus-Domínguez, R. Gómez-Díaz, D. Torres-Lagares, J. R. Corcuera-Flores, J. C. Ruiz-Villandiego, G. Machuca-Portillo, J. L. Gutiérrez-Pérez, M. A. Serrera-Figallo

**Affiliations:** ^1^Oral Surgery Department, Dentistry Faculty, University of Seville, Seville, Spain; ^2^Instituto de Biomedicina de Sevilla, Seville, Spain; ^3^Dentistry in Handicapped Patients Department, Dentistry Faculty, University of Seville, Seville, Spain; ^4^Dentistry in Handicapped Patients Department, Quirón Hospital, San Sebastián, Spain; ^5^Oral and Maxillofacial Unit, Virgen del Rocio Hospital, Seville, Spain

## Abstract

**Aim:**

Aware that Down Syndrome patients present among their clinical characteristics impaired immunity, the aim of this study is to identify the statistically significant differences in inflammation-related gene expression by comparing Down Syndrome patients with Periodontal Disease (DS+PD+) with Down Syndrome patients without Periodontal Disease (DS+PD-), and their relationship with periodontitis as a chronic oral inflammatory clinical feature.

**Materials and Methods:**

Case study and controls on eleven Down Syndrome patients (DS+PD+ vs. DS+PD-). RNA was extracted from peripheral blood using a Qiagen PAXgene Blood miRNA Kit when performing an oral examination. A search for candidate genes (92 selected) was undertaken on the total genes obtained using a Scientific GeneChip® Scanner 3000 (Thermo Fisher Scientific) and Clariom S solutions for human, mouse, and rat chips, with more than 20,000 genes annotated for measuring expression levels.

**Results:**

Of the 92 inflammation-related genes taken initially, four genes showed a differential expression across both groups with a *p* value of <0.05 from the data obtained using RNA processing of the patient sample. Said genes were TNFSF13B (*p* = 0.0448), ITGB2 (*p* = 0.0033), ANXA3 (*p* = 0.0479), and ANXA5 (*p* = 0.016).

**Conclusions:**

There are differences in inflammation-related gene expression in Down Syndrome patients when comparing patients who present a state of chronic oral inflammation with patients with negative rates of periodontal disease.

## 1. Introduction

Down Syndrome covers a large number of pathologies affecting practically every bodily system or apparatus, including cardiovascular; haematological; skeletal; muscular; nervous; endocrine; ear, nose, and throat; ocular; and digestive. It affects to a large extent their oral health and, accordingly, their dental treatment. Periodontal diseases such as necrotizing gingivitis, periodontitis, and aggressive periodontitis are among the most common oral complications that usually affect this type of patients due to their high incidence. Aggressive periodontitis is particularly serious given that patients tend to lose almost all their teeth during bouts recurring every 7-9 years [1].

Although aggressive periodontitis shows characteristic clinical features similar to those of the population who do not suffer from the syndrome, special features in periodontal pathogens have been identified, such as an increase in *Porphyromonas gingivalis* (Pg) and *Aggregatibacter actinomycetemcomitans* (Aac) in these patients compared to patients who do not suffer from trisomy of chromosome 21 (T-21) but who do present aggressive periodontitis [2]. Other species, such as *Fusobacterium nucleatum* (Fn), *Prevotella nigrescens*, or *Actinomyces naeslundii*, are also present in these individuals more often than in those who do not suffer from T-21 [3]. There is speculation about the importance of a particular Pg clone in the triggering of this type of periodontitis (the Pg-JP2 clone) or superinfection by herpes virus, including Epstein-Barr or cytomegalovirus [4, 5].

However, what appears to take on increasing importance in this disease is host response and individual susceptibility, since, in every case of aggressive periodontitis a delay occurs in the chemotactic response of the neutrophils, a specially impaired response in patients with this syndrome [6]. It is known that Down Syndrome patients have many immunological impairments which take part in the development of periodontal diseases, with numerous biomolecular impairments having been identified [7–13].

In this study, we have attempted to identify the statistically significant difference in inflammation-related gene expression by comparing patients with Down Syndrome with Periodontal Disease (DS+PD+) with patients with Down Syndrome without Periodontal Disease (DS+PD-). In this way, the aim is to show the relationship of periodontitis as a chronic oral inflammation with some of the 92 reference genes obtained from the results of the work of another research group [14] and which were obtained from quantifying RNA samples from six Down Syndrome children using TaqMan™ Array Plate Human Inflammation Kit [15].

## 2. Materials and Methods

A retrospective study was carried out on cases and controls which was approved by the Virgen del Rocío Hospital Ethics Committee. Patients or their corresponding guardians provided their consent given that the benefits are direct for the research patient.

### 2.1. Sample and Groups

The study groups are (1) Down Syndrome patients without Periodontal Disease (DS+PD-) and (2) Down Syndrome patients with Periodontal Disease (DS+PD+). The patients in the control group had not been diagnosed with periodontal disease (PD) at any time in their lives and had no bleeding to the catheter (BOP). The patients in the study group had been diagnosed with PD, although when a restoration with implants had to be carried out, the disease was inactive, or the patient had previously lost all his teeth. The criteria used to determine that a patient suffered from PD was the one developed by Bassani et al. [16]. According to this criterion, PD is defined by the presence of 3 or more teeth with 1 or more sites with loss of clinical attachment level (CAL) greater than or equal to 3 mm (1) adding the existence of BOP in the mentioned sites.

The exclusion criteria are as follows: non-Down Syndrome patients, patients receiving treatment which could potentially affect bone metabolism, and patients with active periodontal disease or untreated. The existence or not of periodontal disease was confirmed using the patients' clinical records. Seven patients not suffering from the disease were included compared to four patients who did, thus making a total of eleven patients in the study. Demographic and clinical variables were taken from medical records.

### 2.2. Sampling and Total RNA Isolation

At the time when the patients who were selected and included were examined, a sample was taken comprising two blood collections from the medial cubital vein per person in PAXgene™ tubes (5 ml), reference 762165 (100 tubes), with the final aim of extracting RNA. Transfer of the samples to the processing centre was undertaken under refrigerated conditions (2-8°C) and never took more than three days.

RNA sample extraction was performed using a Qiagen PAXgene Blood miRNA Kit reference 763134 at a QIAcube automated station. Subsequently, a database was built for the samples which detailed, inter alia, RNA quantification data.

First, RNA concentrations were quantified using visible light spectroscopy employing NanoDrop 2000c equipment (Thermo Fisher Scientific) to ensure the correct processing of these before storage. Second, a much more precise measurement was taken, using fluorescence and using Qubit 3.0 equipment (Thermo Fisher Scientific) to study gene expression, the results of which were added to the database.

### 2.3. Functional Analysis of Expressed Genes

The RNA selected were amplified and hybridized using the “GeneChip® WT PLUS Reagent Kit” (Thermo Fisher Scientific, Santa Clara, CA, USA). Amplification was carried out from a total of 55 nanograms of initial RNA, and then the procedures described in the “GeneChip® WT PLUS Reagent Kit” were followed.

Amplification of the cDNA was quantified, fragmented, marked, and prepared for hybridization from the GeneChip® Clariom S Human Array (Thermo Fisher Scientific) for human, mouse, and rat, with more than 20,0000 genes annotated for expression level measurement using 5.5 *μ*g of the product of simple chain cDNA and following the protocols of the GeneChip® Fluidics Station 450 (Thermo Fisher Scientific). Finally, the analysis performed was normalized using the Robust Multiarray Average (RMA) method.

### 2.4. Statistical Analysis from the 96-Plex Card Genes

The analysis of the different gene expression was undertaken using Transcriptome Analysis Console (TAC, Affymetrix) Software. The reference genes taken for the search in our study were those from the results used in a prior study carried out by another group [14] and which include 92 inflammation-related genes and 4 reference genes (Table 1). This other group conducted its experiment from the quantification of the RNA samples of six Down Syndrome children using a TaqMan™ Array Plate Human Inflammation Kit [15]. Values that were lower than 0.05 were deemed statistically significant.

## 3. Results

### 3.1. Gene Expression Analyses

Of the 92 inflammation-related genes selected from a prior study of another group [14] which were searched using Transcriptome Analysis Console (TAC, Affymetrix) Software, four genes showed differential expression across the two groups of patients studied (Down Syndrome patients with Periodontal Disease compared to Down Syndrome patients without Periodontal Disease (*p* < 0.05)). Said genes were ITGB2, TNFSF13B, ANXA3, and ANXA5. Three of them showed a reduced expression, whilst only one of them showed increased expression (Table 2).

### 3.2. Functional Analyses of Differentially Expressed Genes

Each of the four genes that showed differential expression in the patients studied was analysed. The analysis of each of the genes was conducted using the National Center for Biotechnology Information (NCBI) and Online Mendelian Inheritance in Man® (OMIM®) databases. The metabolic pathways of the four genes were also examined using the Kyoto Encyclopaedia of Genes and Genomes (KEGG) database and the reactome for those genes whose information was not available on the KEGG (Table 3).

In accordance with these databases, it was observed that none of the genes share routes, and that for all of them there is a defined participation, except for Annexin 3, for which no information is available concerning participation in cell pathways/metabolic routes.

Conversely, in the “Diseases” section on the KEGG, where disease routes have been collected and represented, each of the genes was searched for, in order to discover whether there was any direct relation with any disease. Only ITGB2 gave a positive result with the clinical features of leukocyte adhesion deficiency, related to the route of transendothelial migration of leukocytes (hsa04670 from KEGG).

## 4. Discussion

Of the 92 inflammation-related genes which were included in our study, four showed statistically significant expression differences across the patients studied (Down Syndrome patients, one of them with positive Periodontal Disease (DS+PD+) compared to negative Periodontal Disease (DS+PD-)). Said genes were TNFSF13B, ITGB2, ANXA3, and ANXA5: three of them showed significantly diminished expression (TNFSF13B, ANXA3, and ANXA5) and one of them showed increased expression (ITGB2). This could be explained by its location, since the latter is the only one of the genes which is found on chromosome 21. The rest have a different cytogenetic location (TNFSF13B on chromosome 13, whilst ANXA3 and ANXA5 on chromosome 4), which seems to corroborate that Down Syndrome causes impairments to the complete genome.

TNFSF13B codifies a cytokine protein of the tumour necrosis factor superfamily, which acts as a ligand for receptors TNFRSF13B/TACI, TNFRSF17/BCMA, and TNFRSF13C/BAFR. TNFSF13B is also known as a lymphocyte B stimulator or B-cell activating factor (BAFF), in reference to its involvement in the activation, proliferation, and differentiation of this type of cell [15]. The impairment of TNFSF13B has been described in autoimmune diseases such as systemic lupus erythematosus [17], Sjögren syndrome [17, 18], and especially, rheumatic diseases [19], although always with significantly higher levels than the healthy controls. BAFF (TNFSF13B) is defined as a function regulator of B and T cells, with proinflammatory and anti-inflammatory effects [15, 18]. In the same way, it is described as necessary for B lymphocyte maturation. Furthermore, the participation of TNFSF13B in the “cytokine-cytokine receptor interaction” cell route reveals other diseases in which gene impairment such as Crohn's disease, DM-I, intestinal inflammatory disease, ulcerative colitis, and juvenile idiopathic arthritis could participate [20]. BAFF (TNFSF13B) improves survival and activation of plasma blasts which involve a greater autoimmune response [21, 22]. It seems inevitable to state that Down Syndrome patients who have a lower expression of TNFSF13B show greater susceptibility to suffering from periodontal disease due to immune and inflammatory deregulation, as well as lower B-cell survival being due to a lower degree of BAFF expression compared to the groups suffering from the syndrome, but not periodontitis.

The beta 2 integrin chain gene (ITGB2) codifies an integrin of the beta chain which combines with other chains to form different integrins, proteins that mediate cell adhesion, as well as cell signalling. Defects in the gene have been related to impairments in the immune system linked to leukocyte adhesion, by affecting the CD18 leukocyte antigen [23]. Already in 1988, it was shown that CD18 was increased in lymphoblastoid cells in Down Syndrome patients [24]. Our results also showed an increase in expression with a fold change of 1.71. ITGB2 is the only gene which is directly related to the disease known as leukocyte adhesion deficiency (LAD), an immunodeficiency which is characterized mainly by the nonmobilization or migration of leukocytes, specifically neutrophils, to lesion sites [25, 26]. Defects associated with beta-2 integrin expression are due to the genetic form I (LAD-1). Patients suffer recurring infections and a delay in the scarring of wounds. Depending on the percentage of leukocytes expressing CD18 on their surface, varying degrees of the diseases may be categorized as mild, moderate, and severe. Gingivitis and periodontitis, with the characteristic of the nonformation of abscesses, may be highlighted among the common infections for LAD-I [26]. The fact that some patients in our study developed periodontal diseases compared to other patients who did not may be explained by the degree of ITGB2 expression on the surface of their leukocytes, despite the fact that both study groups suffered from chromosome 21 trisomy. A possible future research path could be to study whether susceptibility to periodontitis could be determined on analysing CD18 levels in these patients.

ANXA3 and ANXA5 are part of the annexin family. Annexins, basically, are link proteins to calcium-dependent phospholipids. Whilst for the ANXA3 gene there is scarcely any information available in the human genome database [27], Annexin 5 plays an important role in cell signal transduction, inflammation, growth, and differentiation, as well as an anticoagulant and placental protein [28]. The different cell distributions of annexins are very broad. However, there are selective annexins, as in the case of A3 which is only expressed in the myeloid, neutrophil, and macrophage cell lineage, [29, 30]. During the search for information on ANXAS, no relationship was found with any direct human disease by gene impairment, although changes in expression levels do seem to perform a fundamental role in the physiopathology of certain diseases [31]. Nothing has been described for ANXA3, but ANXA5 is related, at least its allelic variant defined in the OMIM® as “Pregnancy loss, recurrent, susceptibility to, 3”, with antiphospholipid autoimmune syndrome, characterized by recurrent thrombosis in macro and microvascularization, giving rise to myocardial infarction, pulmonary embolism, gangrene, recurrent pregnancy loss, and others [31]. ANXA5 promotes endocytosis, regulates tissue factor expression, and participates in protecting against phagocytosis, being capable of binding to gram-negative bacteria and reducing their endotoxin activity [32, 33]. This last point described is extremely interesting, since periodontal pathogenic bacteria are mainly gram-negative bacteria. In a study conducted on pure culture bacteria in the presence of calcium, it has been shown that ANXA5 is capable of binding to the lipid A portion of the lipopolysaccharide (LPS) of gram-negative bacteria, and that like binding to phospholipids, it does so in a very efficient and speedy manner. Similarly, it has been demonstrated that it reduced the effects of the endotoxin of the LPS [32]. In our results, we show how patients who suffer from periodontal disease present ANXA5 expression levels lower than those patients who do not suffer from periodontitis.

In conclusion, this study highlights how despite the two groups of patients presenting the same genetic syndrome, chromosome 21 trisomy, they do not necessarily have to suffer certain immune and/or inflammatory impairments to the same degree. Differential expression of inflammation-related genes when comparing groups of Down Syndrome patients who suffer from periodontitis or not shows that certain patients present greater individual susceptibility to developing inflammatory symptoms, such as periodontitis, which may possibly be explained by impairments to metabolic/cell routes/pathways from impairment of the genes involved.

We must point to the fact that it will be necessary to undertake studies in greater depth for the validation of the expression of the genes identified in this study to be able to confirm the results obtained.

## Figures and Tables

**Table 1:** 92 inflammation-related genes and 4 reference genes.

18Sª	GAPDHª	HPRT1ª	GUSBª	A2M	ADRB1	ADRB2	ALOX12	ALOX5	ANXA1	ANXA3	ANXA5
KLK3	BDKRB1	BDKRB2	CACNA1C	CACNA1D	CACNA2D1	CACNB2	CACNB4	CASP1	CD40	CD40LG	CES1
LTB4R	MAPK14	NR3C1	HPGD	HRH1	HRH2	HTR3A	ICAM1	IL1R1	AL2RA	IL2RB	IL2RG
IL13	ITGAL	ITGAM	ITGB1	KTGB2	KLK1	KLK2	KLKB1	KNG1	LTA4H	LTC4S	MC2R
NFKB1	NOS2	PDE4A	PDE4B	PDE4C	PDE4D	PLA2G1B	PLA2G2A	PLA2G5	PLCB2	PLCB3	PLCB4
PLCD1	PLCG1	PLCG2	MAPK1	MAPK3	MAPK8	PTAFR	PTGDR	PTGER2	PTGER3	PTGFR	PTGIR
PTGIS	PTGS1	PTGS2	TBXA2R	TBXAS1	TNF	TNFRSF1A	TNFRSF1B	VCAM1	IL1R2	PLA2G7	PLA2G10
PLA2G4C	IL1RL1	HTR3B	TNFSF13B	CYSLTR1	HRH3	PLA2G2D	IL1RAPL2	KLK14	PLCE1	KLK15	LTB4R

ªFrom the 96-plex gene card used in the study used as a reference study [14].

**Table 2:** Results of differential gene expression across the two study groups (Down Syndrome patients with Periodontal Disease (DS+PD+) and Down Syndrome patients without Periodontal Disease (DS+PD-)).

Gene	Gene ID-OMIM	Gene name	DS+PD+ AVG (log2)	DS+PD-	DS+PD+ standard deviation	DS+PD- standard deviation	Fold change	*p* value	Chromosome
TNFSF13B	∗ 603969	Tumor necrosis factor ligand superfamily member 13B	11.67	12.66	0.35	1.44	-2	0.0448	Chr13
ITGB2	∗ 600065	Beta 2-integrin	7.79	7.02	0.63	0.16	1.71	0.0033	Chr21
ANXA3	∗ 106490	Annexin 3	7.7	8.85	0.74	0.99	-2.22	0.0479	Chr4
ANXA5	∗ 131230	Annexin 5	11.85	12.65	0.24	0.65	-1.75	0.016	Chr4

**Table 3:** Summary of the cell routes in which each of the statistically significant inflammation-related genes take part in the patients studied.

Gene	Cellular pathways	*p* value	Fold change
TNFSF13B	(1) Cytokine-cytokine receptor interaction (2) NF-kappa B signalling pathway (3) Intestinal immune network for IgA production (4) Rheumatoid arthritis	0.0448	-2

ITGB2	(1) Rap1 signalling pathway (2) Phagosome (3) Hippo signalling pathway (4) Cell adhesion molecules (CAMs) (5) Complement and coagulation cascades (6) Natural killer cell-mediated cytotoxicity (7) Leukocyte transendothelial migration (8) Regulation of actin cytoskeleton	0.0033	1.71

ANXA3	No data	0.0479	-2.22

ANXA5	(1) Release of platelet cytosolic components	0.016	-1.75

## Data Availability

The data used to support the findings of this study are available from the corresponding author upon request.
